# Publisher Correction: H3K4me3 regulates RNA polymerase II promoter-proximal pause-release

**DOI:** 10.1038/s41586-023-05958-0

**Published:** 2023-03-20

**Authors:** Hua Wang, Zheng Fan, Pavel V. Shliaha, Matthew Miele, Ronald C. Hendrickson, Xuejun Jiang, Kristian Helin

**Affiliations:** 1 Cell Biology Program, Memorial Sloan Kettering Cancer Center, New York, NY USA; 2 Center for Epigenetics Research, Memorial Sloan Kettering Cancer Center, New York, NY USA; 3 The Institute of Cancer Research, London, United Kingdom; 4 Biotech Research and Innovation Centre (BRIC), University of Copenhagen, Copenhagen, Denmark; 5 The Novo Nordisk Foundation Center for Stem Cell Biology (Danstem), University of Copenhagen, Copenhagen, Denmark; 6 Microchemistry and Proteomics Core Facility, Memorial Sloan Kettering Cancer Center, New York, NY USA

**Keywords:** Epigenetics, Transcription

Correction to: *Nature* 10.1038/s41586-023-05780-8 Published online 1 March 2023

In the version of this article initially published, the range of units shown along the *x*-axis label of the third panel in Fig. 2e was incorrect, and has been updated to read “2 h, 4 h, 8 h, 24 h, 48 h” from the original “2 h, 8 h, 24 h, 8 h, 48 h”. The errors have been corrected in the HTML and PDF versions of the article.

