# Author Correction: H3K4me3 regulates RNA polymerase II promoter-proximal pause-release

**DOI:** 10.1038/s41586-023-06778-y

**Published:** 2023-10-31

**Authors:** Hua Wang, Zheng Fan, Pavel V. Shliaha, Matthew Miele, Ronald C. Hendrickson, Xuejun Jiang, Kristian Helin

**Affiliations:** 1 Cell Biology Program, Memorial Sloan Kettering Cancer Center, New York, NY USA; 2 Center for Epigenetics Research, Memorial Sloan Kettering Cancer Center, New York, NY USA; 3 The Institute of Cancer Research, London, United Kingdom; 4 Biotech Research and Innovation Centre (BRIC), University of Copenhagen, Copenhagen, Denmark; 5 The Novo Nordisk Foundation Center for Stem Cell Biology (Danstem), University of Copenhagen, Copenhagen, Denmark; 6 Microchemistry and Proteomics Core Facility, Memorial Sloan Kettering Cancer Center, New York, NY USA

**Keywords:** Epigenetics, Transcription

Correction to: *Nature* 10.1038/s41586-023-05780-8 Published online 1 March 2023

In the originally published version of the paper, the bottom photo in Extended Data Figure 8d was a duplicate of the second photo. We have corrected this inadvertent mistake in the HTML and PDF versions of the article. Additionally, to improve readability, we have updated Supplementary Figure 4 to now spread over three pages.

