# Supplementary material for: H3K4me3 regulates RNA polymerase II promoter-proximal pause-release
Source: Nature. 2023 Mar 1;615(7951):339–48. doi: 10.1038/s41586-023-05780-8 (PMC9995272; doi:10.1038/s41586-023-05780-8)
Supplement: Supplementary file 1 — Supplementary Figs. 1–5. [file 41586_2023_5780_MOESM1_ESM.pdf]

nature portfolio

https://doi.org/10.1038/s41586-023-05780-8

Supplementary information

H3K4me3 regulates RNA polymerase II promoter-proximal pause-release

In the format provided by the authors and unedited

nature portfolio

https://doi.org/10.1038/s41586-023-05780-8

Supplementary information

H3K4me3 regulates RNA polymerase II promoter-proximal pause-release

In the format provided by the authors and unedited

Supplementary Information

a

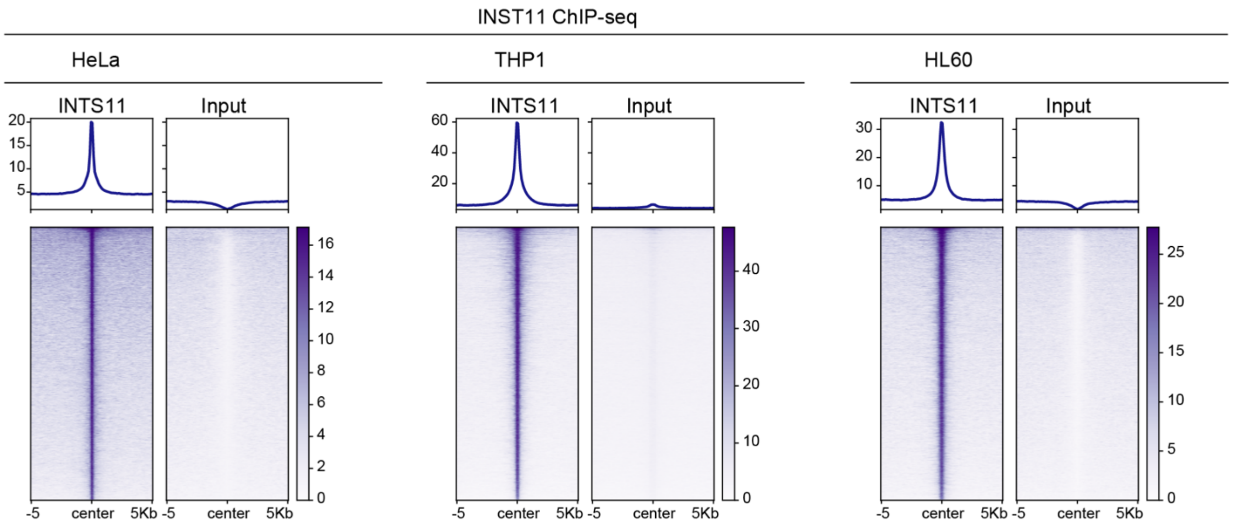

b

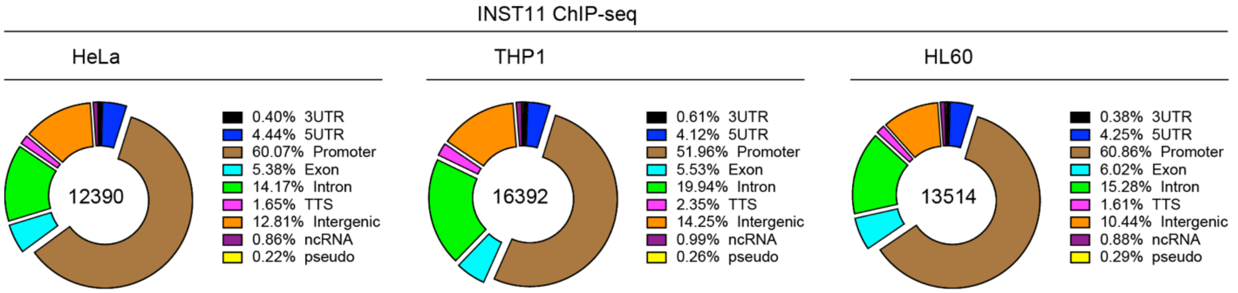

Supplementary Fig.1. INTS11 occupancy at genome wide.

(**a**) INTS11 genomic binding sites as defined as enrichment over input by ChIP-seq.

(**b**) The genomic binding sites of INTS11 were predominantly localized to promoter region of genes.

The INTS11 ChIP-seq data for HeLa, THP1 and HL60 were obtained from published papers.

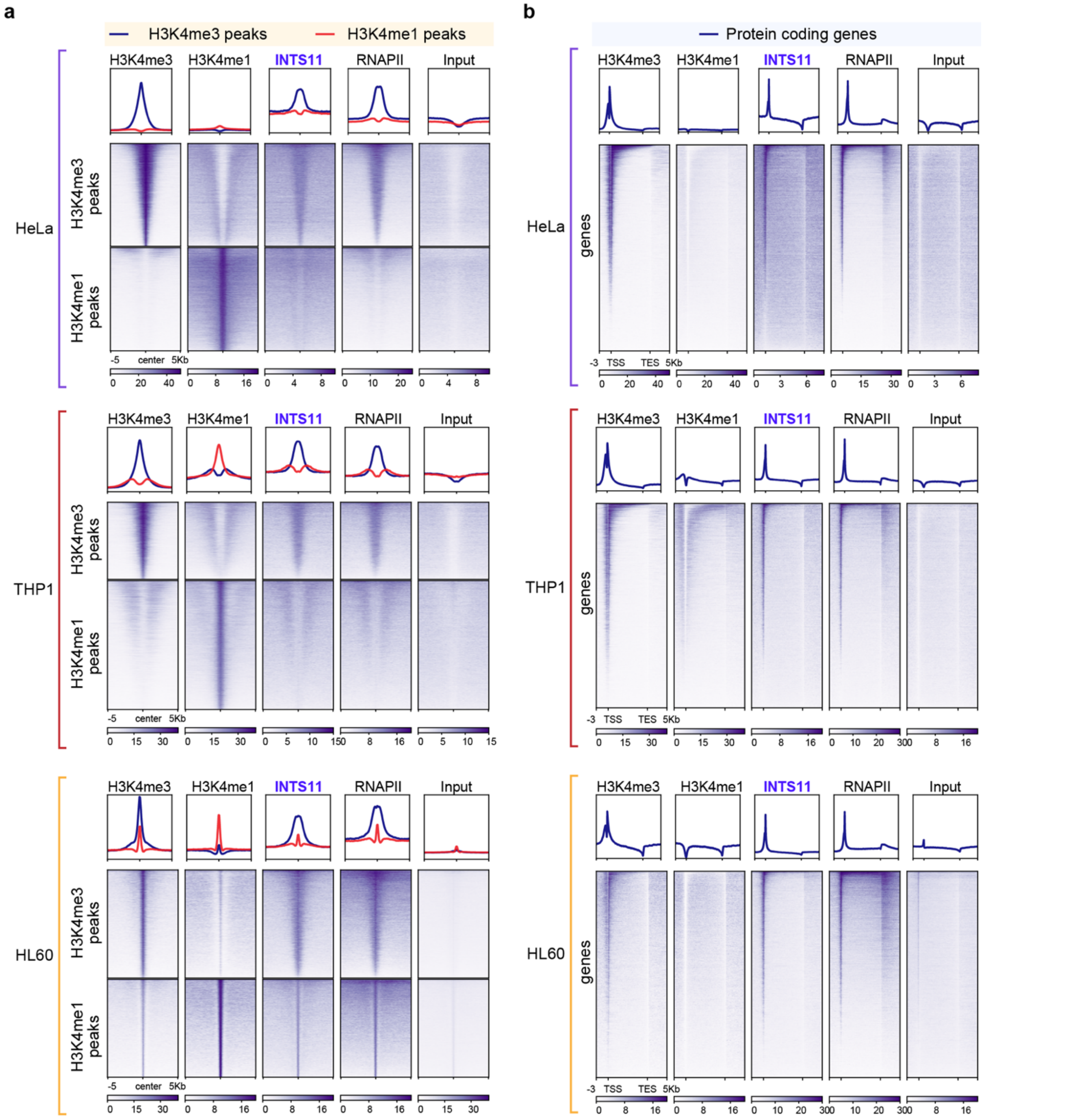

Supplementary Fig.2. INTS11 is enriched at H3K4me3-positive chromatin and binds promoter-proximal region of protein-coding genes.

(**a**) ChIP-seq heatmaps and profiles at H3K4me3 or H3K4me1 peak center in the depicted cell lines. Enrichments were plotted over the enrichment peak center (peak center ± 5 kb).

(**b**) Genome-wide binding averages of ChIP-seq signal on protein-coding genes in different cell lines. The genomic binding sites of INTS11 were predominantly localized to the promoter region of genes. Data are displayed from 3 kb upstream of the transcriptional start site to 5 kb downstream of the end of each gene. TSS, transcription start site, TES, transcription end site. Rows of heatmap are sorted by decreasing H3K4me3 occupancy. The INTS11 ChIP-seq data for HeLa, THP1 and HL60 were obtained from published papers.

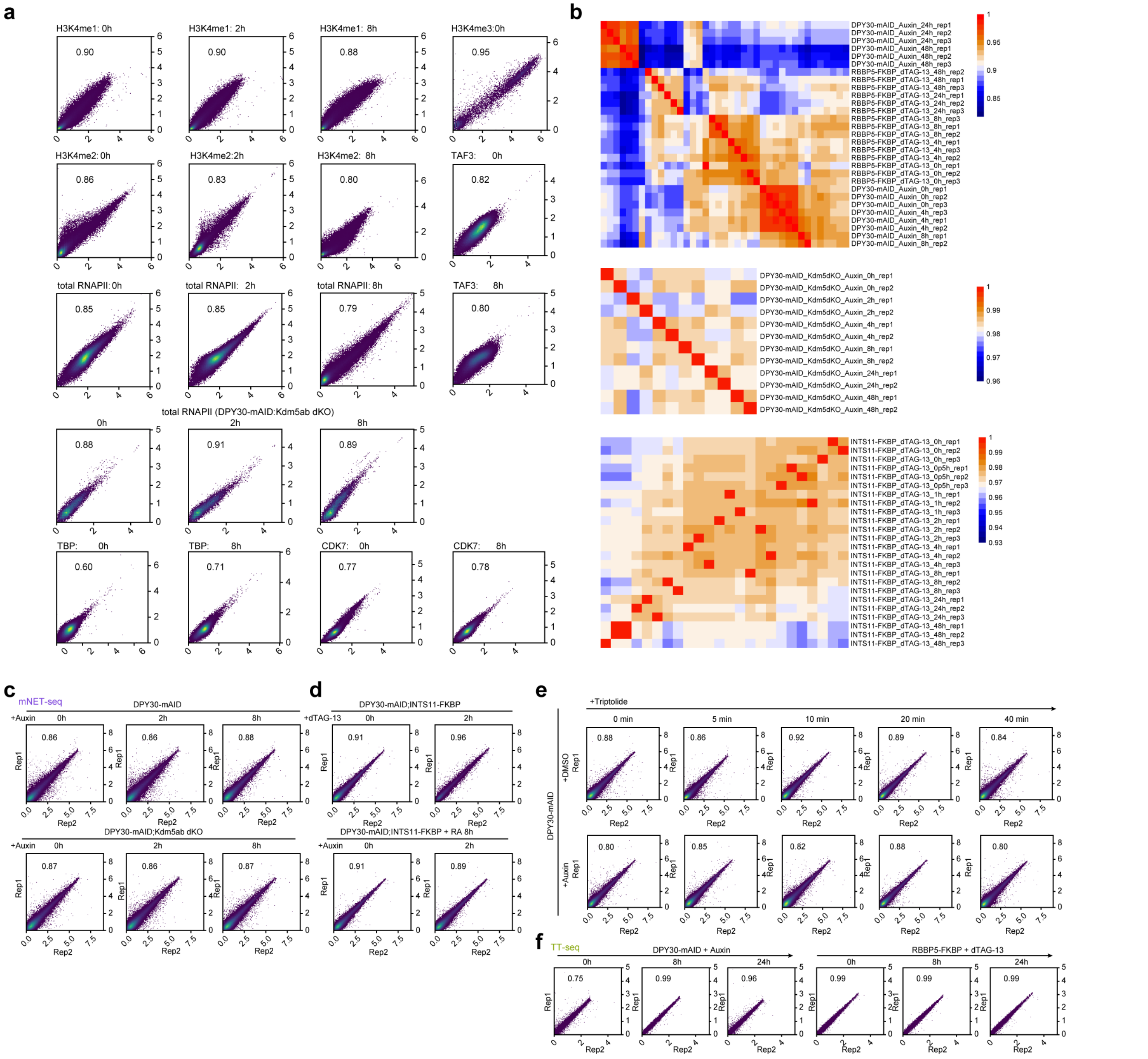

Supplementary Fig.3. Reproducibility of key sequencing data in this study.

(**a**) Correlations between ChIP-seq experiments. Normalized reads per 100 kb bins for DPY30-mAID (*y* axis) and RBBP5-FKBP (*x* axis) degron cells in the indicated samples and time points were plotted. Spearman correlation coefficients are indicated in the panels.

(**b**) Correlations between SLAM-seq experiments. Spearman correlation coefficients are indicated in the panels.

(**c-e**) Correlations between replicates of mNET-seq experiments.

(**f**) Correlations between replicates of TTchem-seq and DRB/TTchem-seq experiments. Normalized reads per 100 kb bins for repeat 1 (*y* axis) and repeat 2 (*x* axis) in the indicated samples and time points were plotted. Spearman correlation coefficients are indicated in the panels.

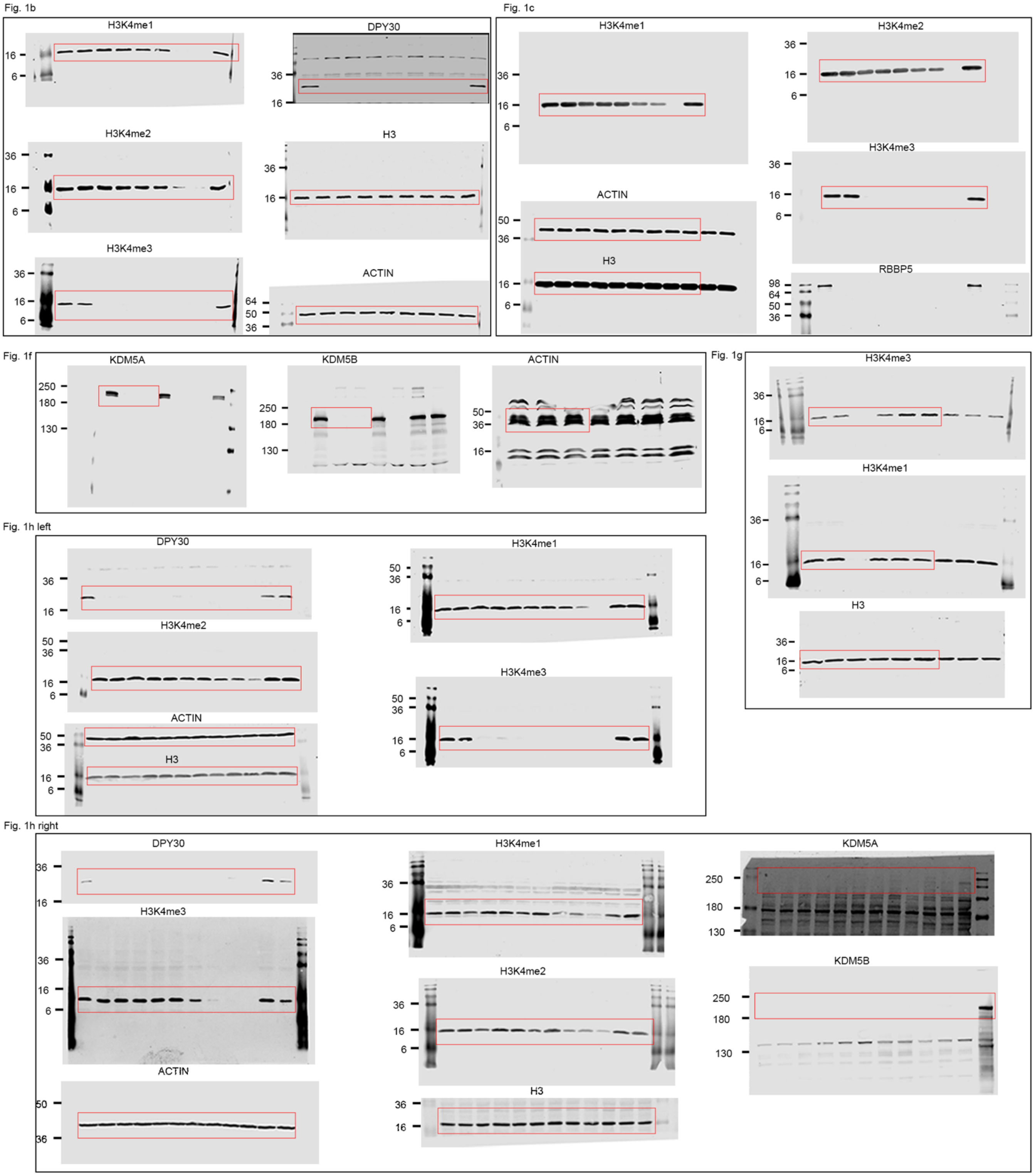

Supplementary Fig.4. Source Data of unprocessed western blots.

Fig. 3a

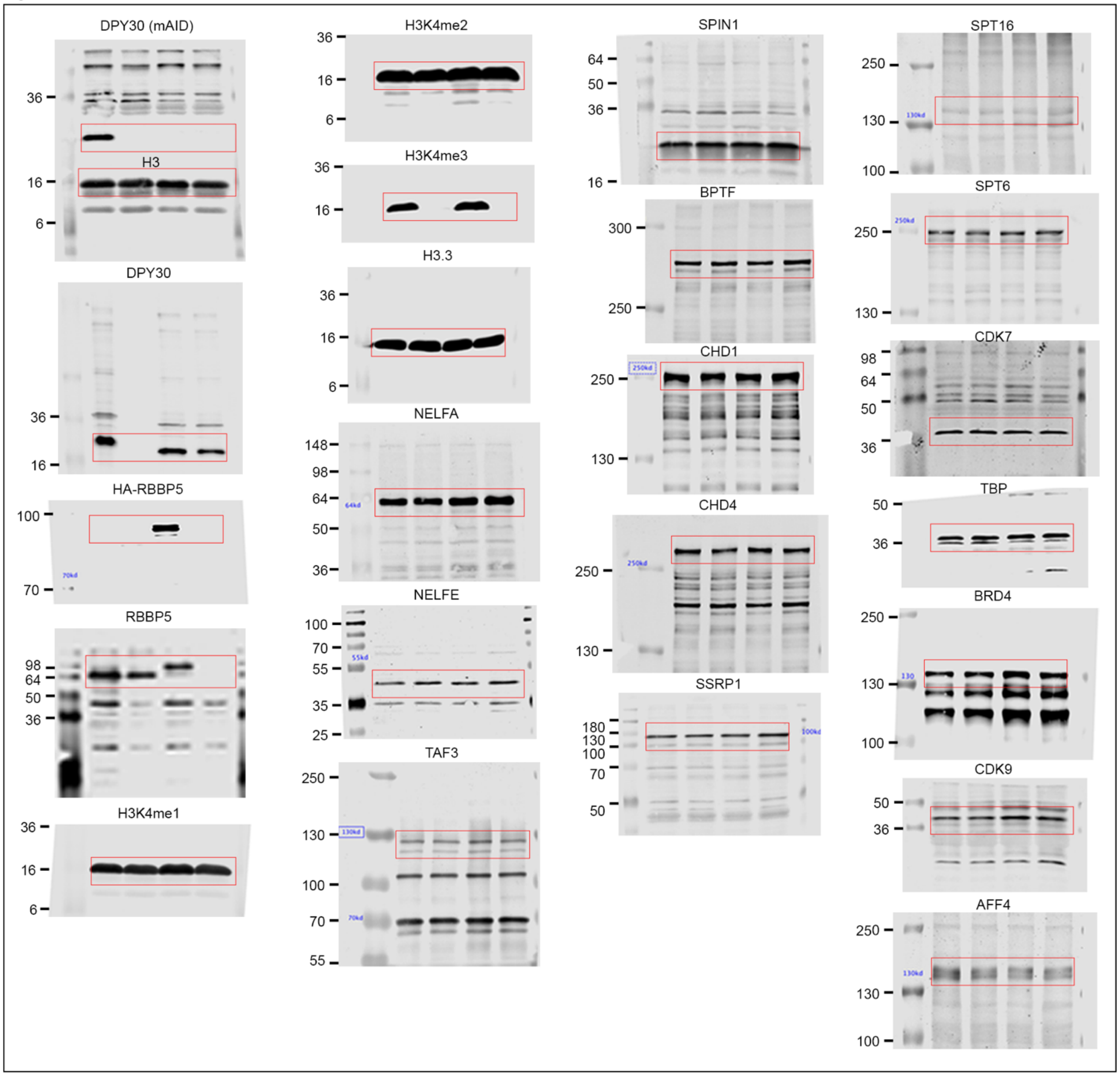

Supplementary Fig.4. Source Data of unprocessed western blots.

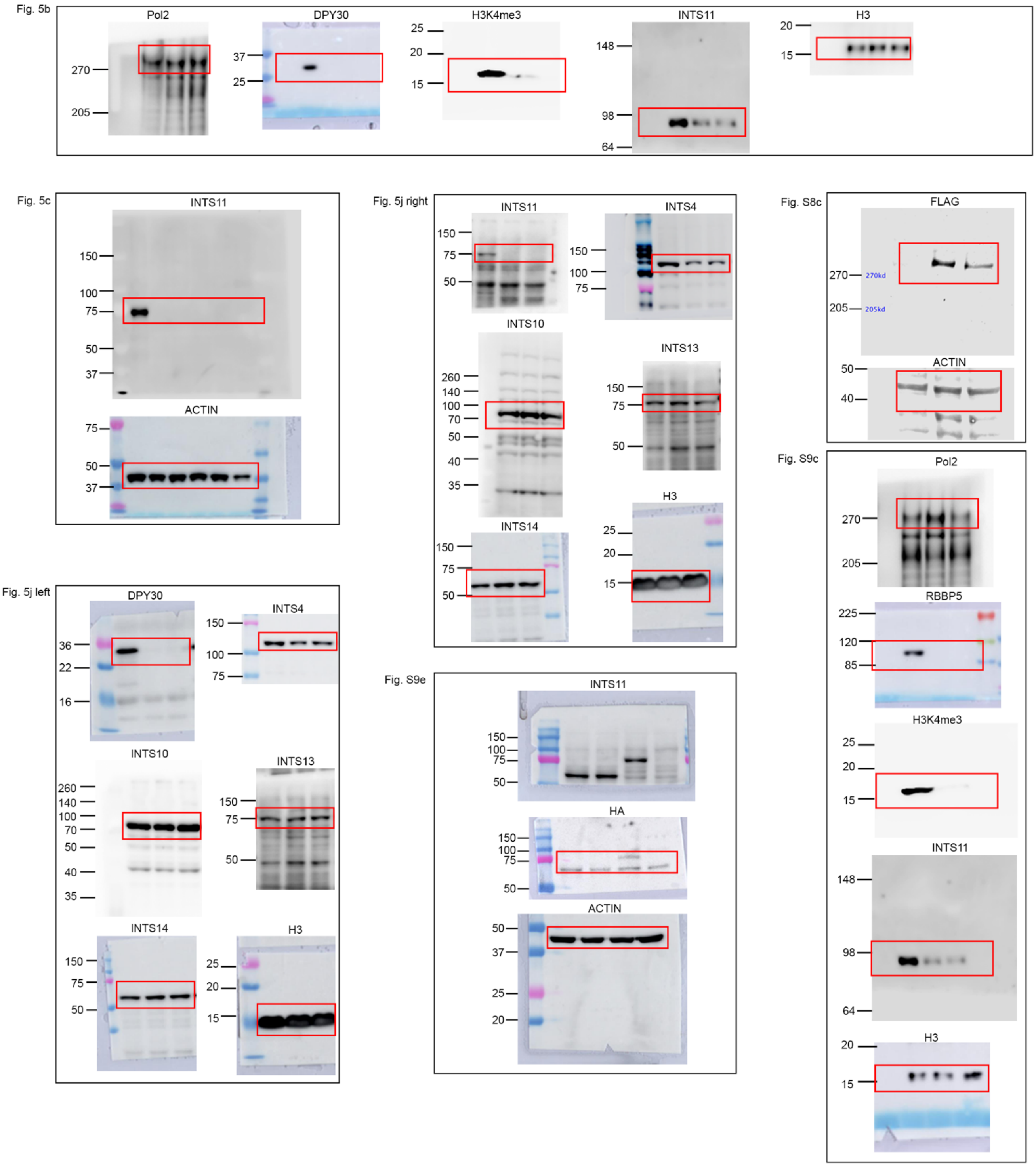

Supplementary Fig.4. Source Data of unprocessed western blots.

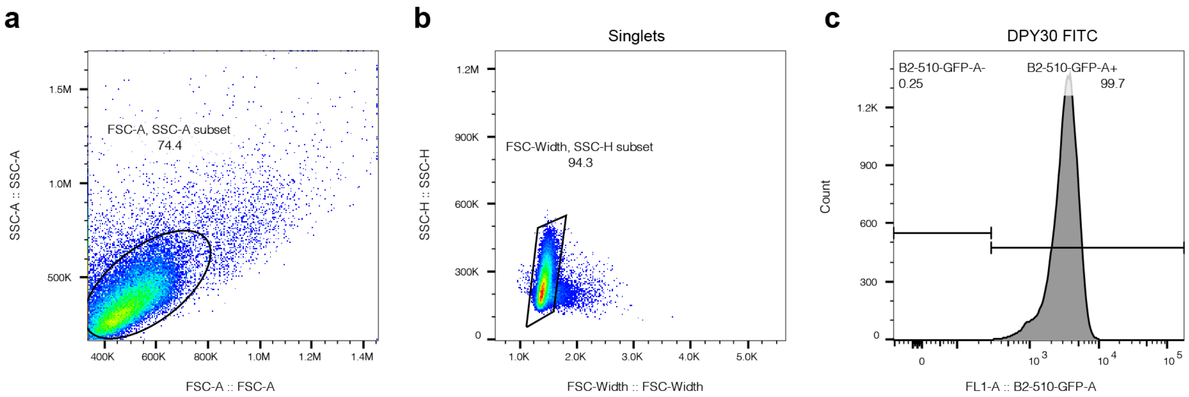

Supplementary Fig.5. Gating strategy for flow cytometry analysis

(**a**) Representative gating for cells in E14 mESCs.

(**b**) Representative gating for singlets in E14 mESCs.

(**c**) Representation of DPY30-positive cells in E14 mESCs.
