# Supplementary material for: H3K4me3 regulates RNA polymerase II promoter-proximal pause-release
Source: Nature. 2023 Mar 1;615(7951):339–48. doi: 10.1038/s41586-023-05780-8 (PMC9995272; doi:10.1038/s41586-023-05780-8)
Supplement: Supplementary file 2 — Reporting Summary [file 41586_2023_5780_MOESM2_ESM.pdf]

nature portfolio

Corresponding author(s): KRISTIAN HELIN

Last updated by author(s): Dec 22, 2022

Reporting Summary

Nature Portfolio wishes to improve the reproducibility of the work that we publish. This form provides structure for consistency and transparency in reporting. For further information on Nature Portfolio policies, see our Editorial Policies and the Editorial Policy Checklist.

Statistics

For all statistical analyses, confirm that the following items are present in the figure legend, table legend, main text, or Methods section.

n/a	Confirmed	
☐	☒	The exact sample size (n) for each experimental group/condition, given as a discrete number and unit of measurement
☐	☒	A statement on whether measurements were taken from distinct samples or whether the same sample was measured repeatedly
☐	☒	The statistical test(s) used AND whether they are one- or two-sided<br>*Only common tests should be described solely by name; describe more complex techniques in the Methods section.*
☐	☒	A description of all covariates tested
☐	☒	A description of any assumptions or corrections, such as tests of normality and adjustment for multiple comparisons
☐	☒	A full description of the statistical parameters including central tendency (e.g. means) or other basic estimates (e.g. regression coefficient) AND variation (e.g. standard deviation) or associated estimates of uncertainty (e.g. confidence intervals)
☐	☒	For null hypothesis testing, the test statistic (e.g. F, t, r) with confidence intervals, effect sizes, degrees of freedom and P value noted<br>*Give P values as exact values whenever suitable.*
☒	☐	For Bayesian analysis, information on the choice of priors and Markov chain Monte Carlo settings
☒	☐	For hierarchical and complex designs, identification of the appropriate level for tests and full reporting of outcomes
☐	☒	Estimates of effect sizes (e.g. Cohen's d, Pearson's r), indicating how they were calculated

Our web collection on statistics for biologists contains articles on many of the points above.

Software and code

Policy information about availability of computer code

Data collection	Real Time quantitative PR data was collected on QuantStudio 6 Flex Real-time PR System v1.3 (Applied Biosystems). Next-generation sequencing data were collected via Illumina sequencing platforms (NextSeq 550). NextSeq550 control software (2.2.0) were used for high-throughput sequencing data collection.
Data analysis	The following software was used for data analysis: GraphPad Prism (v8.0.1) for general statistical analysis and graphing; FlowJo (v10.7.1) for flow cytometry analysis; Enrichr (v.2.1) for gene ontology (GO) enrichment; bcl2fastq (v2.19.0.316), FastQC (v0.11.8), Bowtie2 (v2.4.1), SAMtools (v1.10) , DeepTools (v3.3.0), HISAT (v2.2.1), SlamDunk (v0.3.4 16), DESeq2 (v1.34.0), cutadapt (v3.5) and STAR (v2.7.9a) for genomics data analysis; Proteome Discoverer (v3.1), MSstatsTMT (v2.4.0) and MaxQuant (v1.5.3.30) for proteomics data analysis.

For manuscripts utilizing custom algorithms or software that are central to the research but not yet described in published literature, software must be made available to editors and reviewers. We strongly encourage code deposition in a community repository (e.g. GitHub). See the Nature Portfolio guidelines for submitting code & software for further information.

Data

Policy information about availability of data

All manuscripts must include a data availability statement. This statement should provide the following information, where applicable:

- Accession codes, unique identifiers, or web links for publicly available datasets
- A description of any restrictions on data availability
- For clinical datasets or third party data, please ensure that the statement adheres to our policy

All related raw sequencing and related processed data are deposited and available from the Gene Expression Omnibus (GEO) under the accession numbers GSE181714.

Field-specific reporting

Please select the one below that is the best fit for your research. If you are not sure, read the appropriate sections before making your selection.

☒ Life sciences ☐ Behavioural & social sciences ☐ Ecological, evolutionary & environmental sciences

For a reference copy of the document with all sections, see nature.com/documents/nr-reporting-summary-flat.pdf

Life sciences study design

All studies must disclose on these points even when the disclosure is negative.

Sample size	No specific statistical measure was taken to decide sample size. Minimum sample sizes were predetermined from power estimates based on pilot experiments.
Data exclusions	No data has been excluded from analysis.
Replication	All attempts at replication were successful. Figure legends state how many times each experiment was performed.
Randomization	We did not carry out any randomization because this is either irrelevant or not applicable to this study.
Blinding	Blinding was not done since this study relies on the investigator studying differences in cell lines.

Reporting for specific materials, systems and methods

We require information from authors about some types of materials, experimental systems and methods used in many studies. Here, indicate whether each material, system or method listed is relevant to your study. If you are not sure if a list item applies to your research, read the appropriate section before selecting a response.

Materials & experimental systems

n/a	Involved in the study	
☐	☒	Antibodies
☐	☒	Eukaryotic cell lines
☒	☐	Palaeontology and archaeology
☒	☐	Animals and other organisms
☒	☐	Human research participants
☒	☐	Clinical data
☒	☐	Dual use research of concern

Methods

n/a	Involved in the study	
☐	☒	ChIP-seq
☐	☒	Flow cytometry
☒	☐	MRI-based neuroimaging

Antibodies

Antibodies used

anti-FLAG M2 Affinity gel SIGMA #A2220 50μl for each IP
anti-mini-AID-tag MBL #M2140-3 1:500 for WB
beta-ACTIN Abcam #ab6276 1:10000 for WB
BPTF Abcam #ab72036 1:1000 for WB
BRD4 Abcam #ab128874 1:1000 for WB; 2μg for ChIP
CDK7 Santa Cruz #sc-7344 1:500 for WB
CDK9 Santa Cruz #sc-13130 1:500 for WB; 2μg for ChIP
CHD1 Santa Cruz #sc-271626 1:500 for WB
CHD4 Abcam #ab240640 1:1000 for WB
DPY30 Bethyl Laboratories #A304-296A 1:1000 for WB; 2μg for ChIP
H3 Abcam #ab1791 1:10000 for WB

H3K36me3 Active Motif #61022 1:2000 for WB; 1µg for ChIP
H3K4me1 Cell Signalling #5326S 1:2000 for WB; 1µg for ChIP
H3K4me2 Cell Signalling #9725S 1:2000 for WB; 1µg for ChIP
H3K4me3 Cell Signalling #9751S 1:2000 for WB; 1µg for ChIP
HA-tag (C29F4) Cell Signaling #3724S 1:1000 for WB; 2µg for ChIP
HEXIM1 Cell Signaling #12604S 1:1000 for WB; 2µg for ChIP
INTS11 Atlas Antibodies #HPA029025 1:2000 for WB
KDM5A Cell Signaling #3876T 1:1000 for WB; 2µg for ChIP
KDM5B Helin Lab. Home made #Dain78 1:5000 for WB; 2µg for ChIP
NELFA Santa Cruz #sc-365004 1:500 for WB; 2µg for ChIP
NELFE Santa Cruz #sc-377052 1:500 for WB
PAF1 Abcam #ab137519 1:1000 for WB
Pol II Santa Cruz #sc-899; Abcam #ab817; Cell Signaling #2629S 1:1000 for WB; 2µg for ChIP
Pol II (phospho S2) Active motif #61984 1:1000 for WB; 2µg for ChIP
Pol II (phospho S5) Abcam #ab5131 1:1000 for WB; 2µg for ChIP
RBBP5 Bethyl Laboratories #A300-109A 1:1000 for WB; 2µg for ChIP
SPIN1 Abcam #ab118784 1:1000 for WB
SPT16 Santa Cruz #sc-377028 1:500 for WB
SPT6 Cell Signaling #15616S 1:1000 for WB
SSRP1 Cell Signaling #13421 1:1000 for WB
TAF3 Abcam #ab188332; EMD Millipore #07-1802 1:1000 for WB
INTS11 Atlas Antibodies AB #HPA029025 1:1000 for WB
TFIID (TBP) Santa Cruz #sc-421 1:500 for WB
FITC-conjugated goat α-Rabbit IgG antibody Invitrogen #F-2765 1:500 for Flow

Validation

Antibodies were validated by manufacturers or validated in previous studies. Statements on antibody validation are present on the manufacturer's websites along with relevant references. Additional validation was done by the use of negative control (control IgG) and control cells for Flow Cytometry analysis.

Eukaryotic cell lines

Policy information about cell lines

Cell line source(s)

E14 mESCs: DPY30-mAID:OsTiR1, DPY30-mAID:OsTiR1_Kdm5dKO, DPY30-mAID:OsTiR1_H3.3dKO, RBBP5-FKBP, DPY30-mAID:OsTiR1_RPB1-APEX2, RBBP5-FKBP_RPB1-APEX2

Authentication

Cells were not authenticated

Mycoplasma contamination

Cell lines were tested negative for mycoplasma

Commonly misidentified lines
(See ICLAC register)

No commonly misidentified cell lines were used

ChIP-seq

Data deposition

☒ Confirm that both raw and final processed data have been deposited in a public database such as GEO.

☒ Confirm that you have deposited or provided access to graph files (e.g. BED files) for the called peaks.

Data access links
May remain private before publication.

All related raw sequencing and related processed data are deposited and available from the Gene Expression Omnibus (GEO) under the accession numbers GSE181714.

Files in database submission

GSE181686 mNET-seq on COMPASS-degron cells
GSE181708 Quant-seq on COMPASS-degron cells
GSE181712 TT-seq on COMPASS-degron cells
GSE181892 ChIP-seq on COMPASS-degron cells

Genome browser session
(e.g. UCSC)

https://www.ncbi.nlm.nih.gov/geo/download/?acc=GSE181714&format=file

Methodology

Replicates

ChIP-seq was performed as individual replicates of two biologically independent degron systerms (Auxin and dTAG) mESCs. Information on reproducibility of the technique and antibody performance as described in the general section on replication of methods. Two replicates for mNET-seq and TT-seq.

Sequencing depth

The sequencing was performed in a NextSeq550. The sequencing read information are available as part of the GEO submission (GSE181714).

Antibodies

Target Source/Cat No. Application
BRD4 Abcam #ab128874 1:1000 for WB; 2µg for ChIP
CDK9 Santa Cruz #sc-13130 1:500 for WB; 2µg for ChIP

	DPY30 Bethyl Laboratories #A304-296A 1:1000 for WB; 2µg for ChIP<br>H3K36me3 Active Motif #61022 1:1000 for WB; 1µg for ChIP<br>H3K4me1 Cell Signalling #5326S 1:1000 for WB; 1µg for ChIP<br>H3K4me2 Cell Signalling #9725S 1:1000 for WB; 1µg for ChIP<br>H3K4me3 Cell Signalling #9751S 1:1000 for WB; 1µg for ChIP<br>HEXIM1 Cell Signaling #12604S 1:1000 for WB; 2µg for ChIP<br>KDM5A Cell Signaling #3876T 1:1000 for WB; 2µg for ChIP<br>KDM5B Helin Lab. Home made #Dain78 1:5000 for WB; 2µg for ChIP<br>NELFA Santa Cruz #sc-365004 1:500 for WB; 2µg for ChIP<br>Pol II Santa Cruz #sc-899; Abcam #ab817; Cell Signaling #2629S 1:1000 for WB; 2µg for ChIP<br>Pol II (phospho S2) Active motif #61984 1:1000 for WB; 2µg for ChIP<br>Pol II (phospho S5) Abcam #ab5131 1:1000 for WB; 2µg for ChIP<br>RBBP5 Bethyl Laboratories #A300-109A 1:1000 for WB; 2µg for ChIP
Peak calling parameters	macs2 with Default parameters
Data quality	Data quality was assessed using fastqc
Software	bcl2fastq (v2.19.0.316)<br>FastQC (v0.11.8)<br>Bowtie2 (v2.4.1)<br>SAMtools (v1.10)<br>DeepTools (v3.3.0)

Flow Cytometry

Plots

Confirm that:

☒ The axis labels state the marker and fluorochrome used (e.g. CD4-FITC).

☒ The axis scales are clearly visible. Include numbers along axes only for bottom left plot of group (a 'group' is an analysis of identical markers).

☒ All plots are contour plots with outliers or pseudocolor plots.

☒ A numerical value for number of cells or percentage (with statistics) is provided.

Methodology

Sample preparation	mESCs (1 million) were dissociated with Trypsin/EDTA, resuspended in culture medium, spun, and resuspended in PBS. 0.5 mL of cold Fixation Buffer (BioLegend, 420801) was added and then incubated at room temperature for 10 minutes. Subsequently, the cells were labeled with the unconjugated Rabbit DPY30 antibody (Bethyl Laboratories, A304-296A) and subsequently with a FITC-conjugated goat α-Rabbit IgG antibody.
Instrument	Beckman Coulter CytoFlex.
Software	FlowJo (v10.7.1)
Cell population abundance	Over 20,000 cells were counted for each sample
Gating strategy	The cells were gated using forward and side scatter parameters (FSC/SSC) for singlets (FSC-A/SSC-A). FL1-A :: B2-510-GFP-A is linked to DPY30 expression.

☒ Tick this box to confirm that a figure exemplifying the gating strategy is provided in the Supplementary Information.
